# Supplementary material for: Swelling characteristics and biocompatibility of ionic liquid based hydrogels for biomedical applications
Source: PLoS One. 2020 Apr 20;15(4):e0231421. doi: 10.1371/journal.pone.0231421 (PMC7170238; doi:10.1371/journal.pone.0231421)
Supplement: S1 Data — (PDF) [file pone.0231421.s024.pdf]

Elemental analysis

Elementalanalysis (%) calcd for poly(AAMPSO_3H) MBAA: C 38.59, H 5.87, N 7.61, S16.59; Found: C 40.76, H 5.1, N 6.6, S 16.21.

Elementalanalysis (%) calcd for poly(AE-SO_3) MBAA: C 28.05, H 3.30, N 0.36, S 14.40; Found: C 30.03, H 2.91, N 1.39, S 15,42.

Elementalanalysis (%) calcd for poly(AE-TMA) MBAA: C 50.70, H 8.46, N 7.59; Found: C 45.76, H 8.1, N 6.62.

Elementalanalysis (%) calcd for poly(HPMAA) MBAA: C 56.89, H 8.71, N 11.20; Found: C 55.41, H 8.11, N 8.66.

Elementalanalysis (%) calcd for poly(MAEDMA-SO_3) MBAA: C 46.38, H 7.39, N 5.49, S 12.08; Found: C 50.21, H 7.11, N 6.62, S 13.49.

Elementalanalysis (%) calcd for poly(MAE-SO_3) MBAA: C 32.11, H 4.04, N 0.36, S 13.80; Found: C 31.41, H 6.11, N 2.62, S 12.02.

Elementalanalysis (%) calcd for poly(MAE-TMA) MBAA: C 53.14, H 8.87, N 7.10; Found: C 50.89, H 8.90, N 6.18.

Elementalanalysis (%) calcd for poly(VEtImBr) MBAA: C 42.49, H 5.58, N 13.15; Found: C 41.83, H 7.34, N 12.91.

Elementalanalysis (%) calcd for poly(TMA-VB) MBAA: C 69.16, H 8.70, N 6.98; Found: C 71.41, H 8.11, N 7.62.

Elementalanalysis (%) calcd for poly(MPC) MBAA: C 45.84, H 7.64, N 5.10; Found: C 47.26, H 6.74, N 4.93.

Elementalanalysis (%) calcd for poly(HEMA) MBAA: C 56.46, H 7,88, N 0.36; Found: C 55.67, H 6.29, N 1.42.

Elementalanalysis (%) calcd for poly(MAE-TMA) 700 PEGDA: C 52.11, H 8.73, N 6.61; Found: C 51.73, H 8.90, N 7.72.

NMR of the synthesized monomers

VEtImBr: ^{1}H NMR [250 MHz, acetone-d_6 with a drop of methanol-d_3, δ/ppm relative to tetramethylsilane (TMS)]:10.20 (s, 1H), 8.25 (s, 1H), 8.02 (s, 1H), 7.53 (dd, 1H), 6.17 (dd, 1H), 5.47 (dd, 1H), 4.53 (q, 2H), 1.64 (t, 3H). ^{13}C NMR [250 MHz, acetone-d6,δ]: 206.68, 128.77, 122.98, 119.48, 108.65, 45.23, 14.44.

HPMAA: ^{1}H NMR [250 MHz, $CDCl_3$, δ/ppm relative to tetramethylsilane (TMS)]:1.16 (d, 3H), 1.92 (m, 3H), 3.12 (m, 1H), 3.38 (br, 1H), 3.45 (m, 1H), 3.90 (m, 1H), 5.30 (m, 1H), 5.68 (m, 1H), 6.49 (br, 1H).
